# The Protein Paradox: Elucidating the Complex Nutritional Ecology of the Invasive Berry Pest, Spotted-Wing Drosophila (Diptera: *Drosophila suzukii*)

**DOI:** 10.3389/finsc.2021.787169

**Published:** 2021-12-23

**Authors:** Carrie Deans, William D. Hutchison

**Affiliations:** Department of Entomology, University of Minnesota, St. Paul, MN, United States

**Keywords:** macronutrients, host suitability, microbes, geometric framework, niche

## Abstract

Spotted-wing drosophila (SWD), *Drosophila suzukii*, has become one of the most widely studied insect species over the last decade, largely due to its recent invasion and rapid expansion across the Americas and Europe. Unlike other drosophilid species, which colonize rotting fruit, SWD females possess a serrated ovipositor that allows them to lay eggs in intact ripening fruit, causing significant economic problems for fruit/berry producers worldwide. Though an impressive amount of research has been conducted on SWD's ecology and physiology, aspects of their nutritional ecology remain ambiguous. This review synthesizes the research to date to provide a more comprehensive view of SWD's nutritional relationship with its fruit hosts and associated microbes. Overall, data suggest that SWD's ability to utilize novel resources is likely due to changes in their ecological, rather than physiological, niche that are largely mediated by microbial associations. Studies show that SWD's nutrient intake is comparable to other drosophilid species, indicating limited adaptation to feeding on lower-protein resources. Instead, data show that fruit protein content is a reliable predictor of host suitability and that fruit-microbe dynamics have a strong impact on protein availability. In particularly, fruit protein increases after infestation with SWD-associated microbes, suggesting that initially-suboptimal intact fruits can become protein-rich on a timeframe that is relevant for larval nutrition. This body of work suggests that microbial associations between flies and their fruit hosts can compensate for the nutritional differences between intact and rotting fruit, and that these relationships are likely responsible for SWD's expanded nutritional niche.

## Introduction

Spotted-wing drosophila (SWD), *Drosophila suzukii*, is a fruit fly species endemic to Eastern and Southeastern Asia (1, 2) but is a recent invasive species in North and South America and Europe (3–9). First reported in California (10) and Spain in 2008 (8), SWD quickly spread across North America and Europe by 2013 (11, 12). Belonging to the *D. suzukii* subgenus Sophophora within *D. melanogaster* species group, SWD can be distinguished from most other drosophilids by two morphological features: the presence of dark spots on the wings of males and a large serrated ovipositor on the females. The serrated ovipositor is an ecologically-significant trait, as it allows female SWD to lay eggs in ripening intact fruit, which is a unique niche among other drosophilids that typically lay eggs in split and/or rotting fruit (11, 13, 14). Spotted-wing drosophila is highly polyphagous and is documented to feed on over 25 different plant families, including many economically-important berry and stone fruit varieties (15). Losses can be caused by direct injury to the fruit or by other indirect effects, such as reductions in wine quality based on the spread of *Acetobactor* bacteria to wine grapes by SWD (16). In Minnesota raspberries alone, SWD is estimated to cause more than $2 million in annual losses (17). Additional economic impacts are also incurred through costs associated with prevention and control (14, 18, 19).

**Figure 1:**
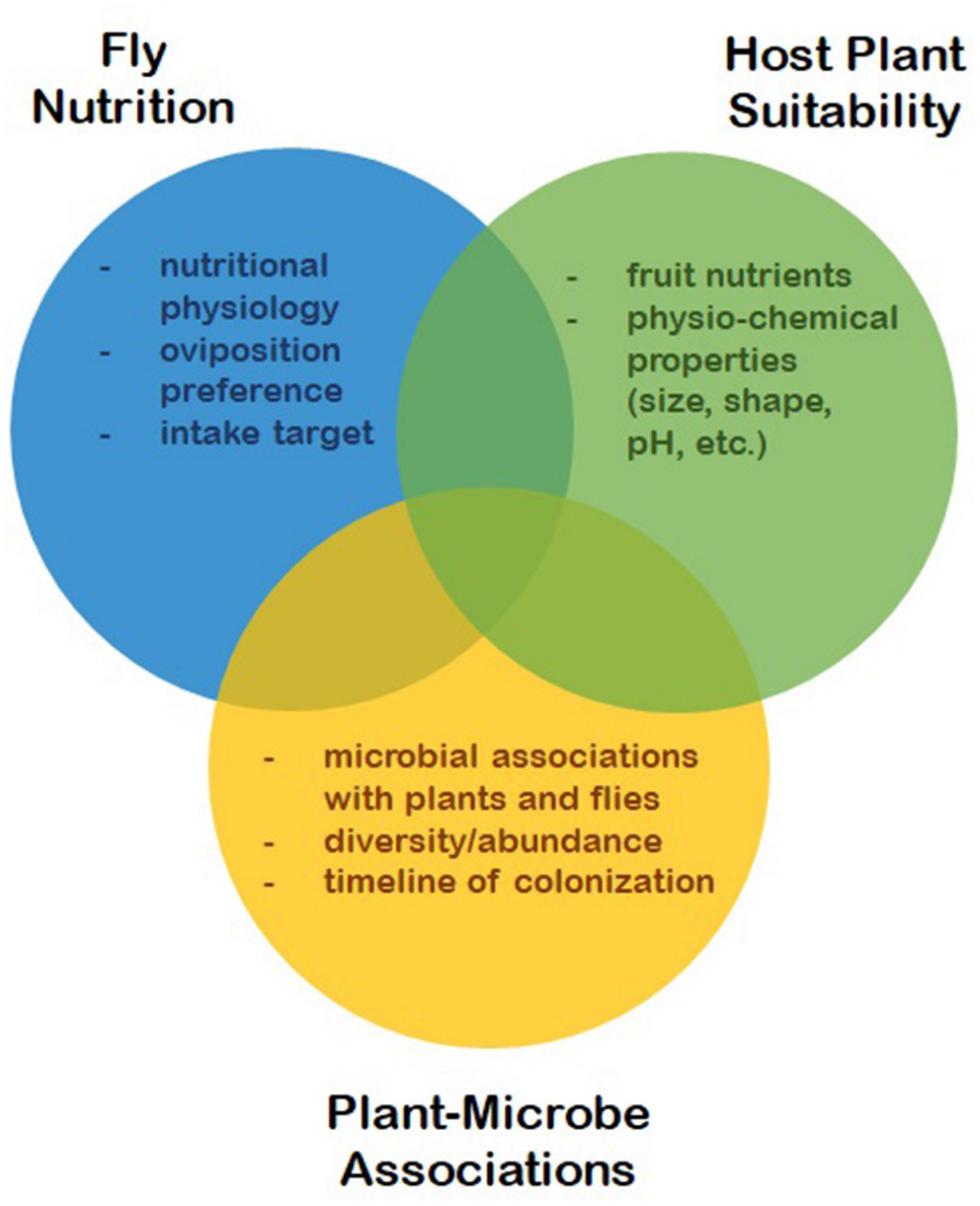
The three main features of *D. suzukii* nutritional ecology and the key characteristics that interact to affect fly fitness.

The nutritional ecology of SWD is rather complex due to its dependence, not only on plant hosts, but also on plant-microbe associations. Fruit tissues provide shelter and food for larvae, while microbes colonizing these tissues provide additional and essential sources of protein, sterols, vitamins and minerals for both larvae and adults. Much research has been focused on different aspects of fly nutrition, including their physiological constraints (13, 20–24), the suitability of different host plants (25–33) and microbial associations between different plant hosts, as well as associations with the flies themselves (34–38). The primary features of SWD's nutritional ecology are illustrated in Figure 1, along with the various characteristics that interact to affect fly fitness. This review synthesizes this body of work to build a more comprehensive understanding of SWD's nutritional ecology, particularly as it relates to host range in an evolutionary context. Understanding the nutritional ecology of SWD is essential for elucidating other aspects of its invasion ecology and developing more effective control strategies.

## Fly Nutrition

In order to understand the nutritional ecology of SWD we first have to elucidate its nutritional requirements. Many studies have attempted to do this by testing different fruits, fruit purees, and artificial diets; however, it is difficult to compare across studies due to the many physical and nutritional differences between these diets. The geometric framework of nutrition (GF) offers a useful method for standardizing dietary variables by focusing on the balance of specific nutrients, typically dietary protein and carbohydrates (39–41). These two macronutrients are required in the greatest amounts by all animals, and a plethora of GF studies have shown that they have the strongest impacts on animal fitness and that their intake is also tightly regulated to reach an optimal balance. This is termed an intake target and is represented by a protein-to-carbohydrate ratio (P:C) (39, 41–43). For SWD, two nutrient-regulation studies have been published that utilize a GF, and both focus primarily on larval performance (13, 23). Despite testing different P:C ratios, Young et al. (23) and Silva-Soares et al. (13) showed that larval survival and developmental times were optimal on more protein-biased diets, which ranged in these studies from a P:C ratio of 1:2 to 24:1. Silva-Soares et al. (13) also found that adult female weight and ovariole number were maximized on high-protein larval diets ranging from 1.5:1 to 1:2. Adults in this study were, however, allowed to feed on the experimental diets after they eclosed, making it difficult to clearly separate the effects of larval vs. adult nutrition. Although they did not utilize a GF, Plantamp et al. (44) assayed adult females fed intact fruit, damaged fruit, and sucrose + yeast solutions, and found that protein acquisition in the adult stage is essential for egg development. Females fed a sucrose + yeast solution had significantly more oocytes per ovary than those fed on fruit alone, even fruit that had been decaying for 16 days and most likely had a higher protein content than intact fruit.

One difficulty in studying SWD adult nutrition is the effect that oviposition has on female feeding behavior, in particular the potential to draw females away from optimal feeding sites. An interesting result of the Silva-Soares et al. (13) and Young et al. (23) studies was that despite the optimality of high-protein larval and adult diets, females showed a strong oviposition preference for high-carbohydrate substrates- a trait that has also been observed in other drosophilids (13, 20, 45, 46). While this may seem contradictory, plant-microbe interactions and their impact on larval nutrition may explain much of this behavior and is discussed more thoroughly in section Microbes and Nutrition.

**Figure 2:**
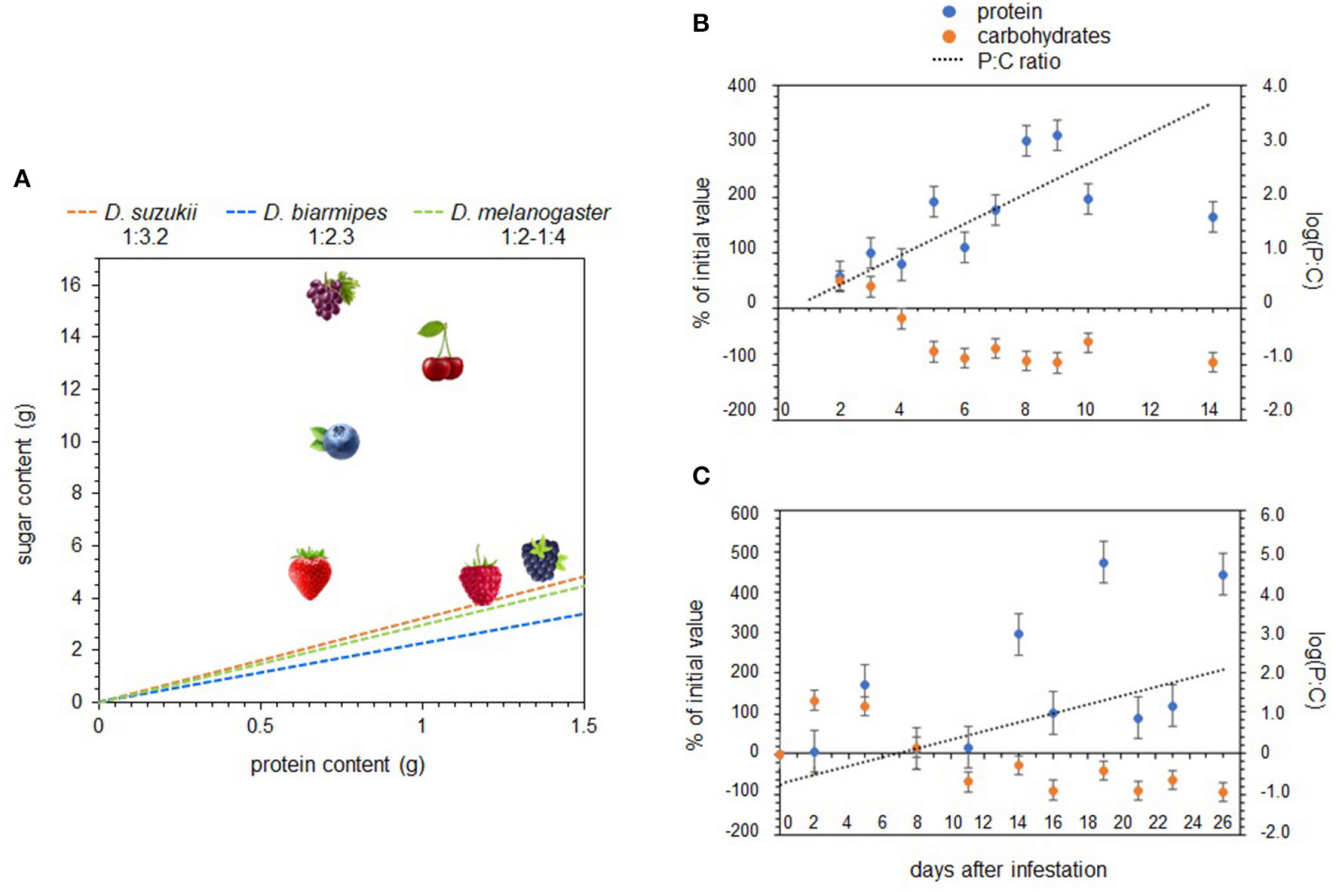
The macronutrient content of different host fruits, as well as the intake targets for *D. suzukii* and two other drosophilid species **(A)**. Macronutrient changes over time in strawberry **(B,C)** after infestation by *D. suzukii*. These graphs were adapted from the raw macronutrient data published in Silva-Soares et al. (13) and Matavelli et al. (48).

Nutrient regulation of specific nutrients has also been used to identify SWD nutritional requirements. Silva-Soares et al. (13) is the only study that has measured nutrient intake in SWD, which was done in larvae using spectrophotometric quantification of food dye. Data showed that SWD larvae strictly regulate protein intake and tolerate a broader range of dietary carbohydrate concentrations. A specific intake target was not explicitly calculated in this study, however, extrapolating from their data suggests that the species average is around 1:3.2. Because SWD feeds on ripening intact fruit with low microbial colonization, it has been hypothesized that SWD larvae may be adapted to lower-protein resources relative to other drosophilid species that feed on microbe-rich rotting fruit. Silva-Soares et al. (13) measured larval nutrient intake for *D. biarmipes*, a closely related species, and the estimated intake target from these data show a species average around 1:2.2, a slightly more protein-rich intake target than SWD. However, other studies looking at nutrient regulation in adult *D. melanogaster* have reported intake targets that are more carbohydrate-biased than SWD, at around 1:4 (46, 47). Additionally, SWD and *D. biarmipes* did not differ significantly in their oviposition preference for low P:C ratio substrates (13). When taken together, these studies do not strongly support the notion that a physiological shift toward more carbohydrate-biased resources has evolved in SWD and rather suggests that SWD's physiological niche largely overlaps with other drosophilid species (Figure 2A) [but see (49)].

## Host Plants Characteristics

Many studies have explored host range (50, 51) and suitability (26, 28–32) for *D. suzukii* by measuring developmental time and adult emergence across different fruits. Fruit purees have also been used to control for differences in the physical characteristics between fruits (skin thickness, fruit morphology, fruit size, etc.). Across laboratory studies, there is quite a strong consensus that raspberries are an optimal host for larval performance, followed by blackberries and strawberries (26, 28–32, 51). The adequacy of blueberries and cherries are less consistent across studies, while grapes consistently produce poor larval performance. Interestingly, the results found across studies that used whole fruits (26, 28, 30–32, 51, 52) and fruit purees (25, 27, 32, 33) are very similar, suggesting that the physical characteristics of different fruits play a minimal role. However, in many of these experiments, larval densities were tightly controlled and because larval competition has significant effects on developmental time and emergence (27), and is likely impacted by structural differences between fruits, this limits our ability to accurately determine the role of physical traits in many of these studies.

It should be noted that others studies have explored the suitability of non-fruit hosts for SWD, including mushrooms and animal manure (22, 53). While these data do show the capacity for SWD to utilize non-fruit under certain circumstances, their relevance to host suitability characteristics or nutrition is limited by the fact that these components were not measured for each. Should future research be able to connect these non-fruit hosts with specific macronutrient profiles or physical characteristics, they could help elucidate the important factors for fly performance.

Combining data on fly nutrition and fruit macronutrient content uncovers some interesting patterns. The most commonly utilized North American fruits, as well as their P:C ratios and energy contents, are listed in Young et al. (23) and summarized in Figure 2A. Raspberries and blackberries represent the most protein-rich fruits, followed by strawberries, cherries, and blueberries, while grapes are the most carbohydrate-biased. Figure 2A shows that the larval intake targets for SWD and two other drosophilid species, *D. biarmipes* and *D. melanogaster*, fall closest to raspberries and blackberries- the two fruits with the greatest SWD suitability (25–28, 30–33, 51, 52). This suggests that the nutritional characteristics of fruit hosts may play a primary role in larval performance and host suitability.

Across 67 fruits, Poyet et al. (51) found that adult emergence was significantly greater in fruits with smaller diameters and those with polydrupes compared to berries or single drupe fruits. Additionally, Little et al. (30) found a significant positive relationship between the number of pupae present on different hosts, fruit brix content (sweetness), and pH, but a negative relationship with firmness. However, in another study focused on boreal fruits, Little et al. (31) found no association between adult emergence, brix content or skin firmness and instead a negative correlation between emergence and pH. Although it appears that fruit pH and morphology, specifically the existence of compartmentalized structures, may play an important role in host suitability, more work is needed to determine exactly how these factors impact larval performance and/or interact with other nutritional characteristics.

Data on the link between oviposition preference and fruit characteristics are less straightforward. Although oviposition preference in whole-fruit and fruit puree choice tests largely supports the preference/performance hypothesis, as oviposition matches the larval suitability data for raspberries and blackberries being the preferred hosts, other studies show discrepancies between suitability and oviposition preference data. Poyet et al. (51) found that female flies generally prefer to lay eggs in large fruits with simple structures (berries or single drupes), black or white coloring, a round vs. oval shape, and rough vs. smooth skin- virtually the opposite characteristics of the most suitable hosts. While Little et al.'s (30) female oviposition data does not match their suitability data, citing a preference for low rather than high pH and low-intermediate rather than high brix ratings, Lee et al.'s (54) oviposition data do, citing an increased likelihood of oviposition in blueberries with high pH and brix ratings. Stewart et al.'s (55) peach data, however, find no effect of firmness or sugar content on oviposition. Additionally, as already discussed, females in GF studies also show an oviposition preference for more carbohydrate-biased diets, with P:C ratios ranging from 1:8 to 1:12. This apparent mismatch between larval suitability and oviposition preference could be affected by many factors and is likely dependent on the host options available, as well as interactions between nutritional and physical factors. However, as the next section will show, the role of microbes in SWD larval nutrition may be key to explaining many of these discrepancies.

## Microbes and Nutrition

So far, we've discussed SWD's nutritional requirements and how it relates to other host plant characteristics. The last, and perhaps most critical, facet of SWD's nutritional ecology is the role that microbes play. Microbes are an essential part of all drosophilid life histories, as they are the primary source of protein, sterols, and other vitamin and minerals for larvae and adults. Microbes also play a significant role as endosymbionts and mediators of chemosensory cues that are important for host selection. These two topics are unfortunately beyond the scope of this review but see (35, 56, 57). Understanding how plant-microbe relationships affect SWD populations is a burgeoning field of study, and elucidating these relationships from a nutritional perspective is essential for understanding SWD's ecology.

The most important microbe in drosophilid nutrition is yeast (13, 23, 27). Plant-yeast associations are widespread in nature, but the most common species found in fruits infested by SWD include: *Hanseniaspora uvarum, Issatchenkia terricola* (formerly *Pichia*), *Rhodotorula mucilaginosa, Candida* spp., *Metschnikowia pulcherrima, Pichia* spp., *Saccharomycopsis vini, Cryptococcus* spp., *Sporobolomyces* spp., *Debaryomyces* spp., *Penicilium paneum*, and *Moniliella megachiliensis* (36, 58). Many of these species are also present in SWD's gut microbiota, including *Hanseniaspora, Issatchenkia*, and *Metschnikowia* species in larvae and adults, and *Candida* species exclusively in larvae (58). Several bacterial species are also associated with wild-caught and laboratory-reared SWD, including *Acetobacter, Enterococcus, Lactococcus, Gluconobacter, Dyella, Orbus, Tatumella*, and *Staphylococcus* species (34, 37). While *Acetobacter* and *Gluconobacter* are known associates of other drosophilid species, SWD's bacteriome is uniquely dominated by *Tatumella*, which is less common in other species. Studies have shown that the abundance and diversity of bacterial species associated with SWD varies throughout the season, between different populations, and across different habitats (37). Bacterial diversity also appears to vary more extensively in adults than larvae (34).

The connection between dietary yeast and SWD performance is well-documented, and has been studied in both presence/absence experiments that utilize anti-microbials in artificial diets (23) and more-detailed studies that incorporate specific yeast strains into artificial diets (36, 38). Young et al. (23) reared larvae on artificial diets that varied in P:C ratio with and without antimicrobials present. The macronutrient profile of the diets containing antimicrobials does not change throughout the experiment because colonization by microbes cannot occur; however, diets without antimicrobials are subject to colonization, and therefore, may change throughout the study. When antimicrobials were present, performance was best on the high-protein diets and worst on the high-carbohydrate diets, however, when antimicrobials were absent, this pattern was reversed such that performance was best on high-carbohydrate diets and worst on the high-protein diets. The fact that larval performance was similar for the high-protein/antimicrobial treatment and the high-carbohydrate/anti-antimicrobial treatment, suggests that the high-carbohydrate diet became a high-protein diet through the opportunistic colonization of microbes as the experiment progressed. However, because microbial growth and/or abundance was not measured in these diets, it is impossible to confirm such a change.

The relationship between host plant nutrients and microbial growth represents a missing link in SWD nutrition. Unfortunately, very few studies that have assessed how microbial growth varies across different fruits and/or how it impacts the macronutrient content of fruit tissues. Silva Soares et al. (13) addressed this issue by monitoring changes in strawberry protein and sugar content after exposure to SWD to see how the macronutrient profiles changed after microbial colonization, and though they did not directly measure microbial abundance on fruit tissue, they did see a significant increase in protein content and simultaneous decrease in sugar content over a 14-day period. Matavelli et al. (48) found a similar pattern with fig macronutrients after colonization by yeast (Figures 2B,C). Because the inoculated fruit tissues did not simply accumulate protein but also become more deplete in carbohydrates, the P:C ratio of fruit changed quite quickly. These results suggest that intact fruits that are sub-optimal high-carbohydrate resources at the time of oviposition can quickly become optimal high-protein resources.

This scenario is made more plausible by data showing that even uninfested intact fruits are not devoid of microbes. Microbes readily colonize split or damaged fruit and are thought to be largely absent on the inside of intact fruit, but Hamby et al. (58) examined yeast community composition in varieties of cherry and raspberry and found between 14 and 20 different yeast species in uninfested fruits. In fact, their data suggest that the microbial dynamics on specific hosts may have greater implications for suitability than the total number of species present. For instance, the total number of yeast species did not differ dramatically between raspberries, an optimal host, and cherries, a less optimal host, but the number of species in infested vs. uninfested fruits was different. A higher number of species was present in infested vs. uninfested raspberries, while more species were actually present in uninfested than infested cherries. The abundance of present yeast species, as measured by colony-forming units, was also greater in infested raspberries than in infested cherries. These patterns of microbial diversity and abundance may explain why raspberries are such optimal hosts for SWD; however, much more data on microbial associations, their growth rates on different fruit hosts, and the impact on host nutrient content are needed.

## Discussion

The synthesis of current research shows that, from a nutritional perspective, the invasion success of SWD is due to interactions between fly physiology, host plant characteristics, and microbial associations that have expanded SWD's ecological niche in such a way that the pest can utilize novel resources. This conclusion is supported by data showing that SWD's nutritional requirements are comparable to other drosophilids and that their intake targets are not drastically more carbohydrate-biased, as expected if they were adapted to low-protein resources like intact fruits (13, 47). Studies have also shown that fruit macronutrient content appears to play a primary role in host suitability, as there is a strong relationship between host optimality and fruit P:C ratio (Figure 2A). While the importance of microbes in fly nutrition is well-established, there are still many unanswered questions about microbial interactions in the field. Despite this, increasing data show that the relationship between microbial diversity/abundance and specific fruit hosts, as well as the microbial dynamics in infested vs. uninfested fruits, impact larval nutrition by turning low-protein intact fruit into high-protein resources on a timescale that is relevant for larval nutrition. This is supported by data showing that fruits can change from low to high P:C tissues rather quickly after initial infestation by SWD (13, 48). Furthermore, the inoculation of intact fruits with fly-associated microbes during oviposition, and subsequently through secondary infections, may expedite this process (59, 60). In fact, the oviposition preference that all drosophilids, including SWD, appear to have for high-carbohydrate substrates is likely driven by the fact that high sugar content fuels microbial growth and likely expedites this transition. Much more work is still needed to support these hypotheses, particularly data on the timeline of microbial colonization of different fruit hosts, it's effect on fruit macronutrients, and the subsequent implications for larval nutrition.

## Author Contributions

CD is responsible for the writing of this article and the creation of the figures. WH is responsible for the editing of the manuscript. CD and WH are responsible for the generation of the concepts and ideas provided. All authors contributed to the article and approved the submitted version.

## Funding

This work was supported by funding from the Agricultural Growth, Research, and Innovation (AGRI) Crops Research Program, of the Minnesota Department of Agriculture, St. Paul, MN (2018-2021), and the Minnesota Agricultural Experiment Station, University of Minnesota, St. Paul, MN, USA.

## Conflict of Interest

The authors declare that the research was conducted in the absence of any commercial or financial relationships that could be construed as a potential conflict of interest.

## Publisher's Note

All claims expressed in this article are solely those of the authors and do not necessarily represent those of their affiliated organizations, or those of the publisher, the editors and the reviewers. Any product that may be evaluated in this article, or claim that may be made by its manufacturer, is not guaranteed or endorsed by the publisher.
